# Improving ambulance coverage in a mixed urban-rural region in Norway using mathematical modeling

**DOI:** 10.1371/journal.pone.0215385

**Published:** 2019-04-12

**Authors:** Pieter L. van den Berg, Peter Fiskerstrand, Karen Aardal, Jørgen Einerkjær, Trond Thoresen, Jo Røislien

**Affiliations:** 1 Department of Technology and Operations Management, Rotterdam School of Management, Erasmus University, Rotterdam, the Netherlands; 2 Department of EMS, Vestfold Hospital Trust, Tønsberg, Norway; 3 Delft Institute of Applied Mathematics, Delft University of Technology, Delft, the Netherlands; 4 Centrum Wiskunde & Informatica, Amsterdam, the Netherlands; 5 Faculty of Health Sciences, University of Stavanger, Stavanger, Norway; 6 Department of Research, Norwegian Air Ambulance Foundation, Oslo, Norway; Central South University, CHINA

## Abstract

**Background:**

Ambulance services play a crucial role in providing pre-hospital emergency care. In order to ensure quick responses, the location of the bases, and the distribution of available ambulances among these bases, should be optimized. In mixed urban-rural areas, this optimization typically involves a trade-off between backup coverage in high-demand urban areas and single coverage in rural low-demand areas. The aim of this study was to find the optimal distribution of bases and ambulances in the Vestfold region of Norway in order to optimize ambulance coverage.

**Method:**

The optimal location of bases and distribution of ambulances was estimated using the Maximum Expected Covering Location Model. A wide range of parameter settings were fitted, with the number of ambulances ranging from 1 to 15, and an average ambulance utilization of 0, 15, 35 and 50%, corresponding to the empirical numbers for night, afternoon and day, respectively. We performed the analysis both conditioned on the current base structure, and in a fully greenfield scenario.

**Results:**

Four of the five current bases are located close to the mathematical optimum, with the exception of the northernmost base, in the rural part of the region. Moving this base, along with minor changes to the location of the four other bases, coverage can be increased from 93.46% to 97.51%. While the location of the bases is insensitive to the workload of the system, the distribution of the ambulances is not. The northernmost base should only be used if enough ambulances are available, and this required minimum number increases significantly with increasing system workload.

**Conclusion:**

As the load of the system increases, focus of the model shifts from providing single coverage in low-demand areas to backup coverage in high-demand areas. The classification rule for urban and rural areas significantly affects results and must be evaluated accordingly.

## Introduction

Emergency Medical Service (EMS) providers are responsible for providing adequate care to out-of-hospital emergency calls. The probability of survival is highly dependent on the response time [1], and it is thus of crucial importance that the distribution of available ambulances allows for rapid responses. In most countries, this importance is stressed by the government by official response time targets. For example, in the Netherlands an ambulance should arrive at the scene within 15 minutes in 95% of cases, while in England the response time target is 8 minutes, which should be met for 75% of all Category A calls [2]. In mixed urban-rural regions different targets for urban and rural areas are common. In Norway, urban and rural areas have response time targets of 12 and 25 minutes, respectively [3]. In order to meet these targets, one must simultaneously optimize for both the location of base stations and the number of ambulances stationed at each base.

A frequently used model for exploring the optimal location of bases for EMS vehicles is the Maximum Covering Location Problem (MCLP) [4]. The model can be used to find the location and necessary number of base stations in order to maximize the coverage given a specific response time target. The model has been used in a wide range of applications [5], such as establishing the optimal location of bases for emergency helicopters in Norway [6,7] and Australia [8] and medical drones in the United States [9,10]. While the MCLP model is suitable for finding locations of base stations, a more advanced model is needed in order to find the optimal number of ambulances at each base. That is, a model that also accounts for the fact that ambulances might be temporarily unavailable with concurrent tasks, such as serving another call. The average fraction of time that an ambulance is busy is called the busy fraction. A generalization of the MCLP model that also incorporates the busy fraction is the more general MEXCLP model [11], allowing for simultaneous optimization of the location of bases and the distribution of available ambulances given the target time threshold. This model has been applied to numerous ambulance location problems [12–14].

The aim of this study was to explore the optimal ambulance distribution in the Norwegian county Vestfold, a county with large urban-rural differences. Using a modified version of the MEXCLP model, exploring a range of different busy fractions, we were able to not only improve significantly on the existing base structure, but also provide simple within-day and within-week optimizations to better utilize the available number of ambulances during day and night, weekday and weekend. This work also provides valuable insights into the behavior of the MEXCLP model in mixed urban-rural regions. Despite the many applications of the MEXCLP model, this has not yet been studied. The results show the trade-off between back-up coverage for high-demand areas and single coverage for low-demand areas and analyses how this trade-off depends on the load of the system. While the study focuses on strategic planning and the determination of a daily schedule for the ambulances, the results can also be used for real-time planning. The model precisely describes the optimal distribution for each number of available ambulances.

## Material and methods

### Setting

The Vestfold region in Norway is situated southwest of the capital Oslo along the Oslo fjord. It covers an area of 2224 km^2^, and in 2017 it had 247 048 inhabitants [15]. The region has one major hospital, the Vestfold Hospital Trust located in Tønsberg, which is responsible for the emergency medical service (EMS) in most of the region. The two municipalities Sande and Svelvik do not fall under the responsibility of the Vestfold Hospital Trust, and were excluded from the analysis. Vestfold Hospital Trust is therefore responsible for an area of 1990 km^2^ with a total of 230 899 inhabitants. The service currently contains five fixed ambulance bases and operates 13 ambulances during the day and eight during nighttime.

### Population and incident data

Population data are freely available from Statistics Norway, and in the descriptive analyses of the region we use data from January 1, 2017 [15].

**Fig 1:**
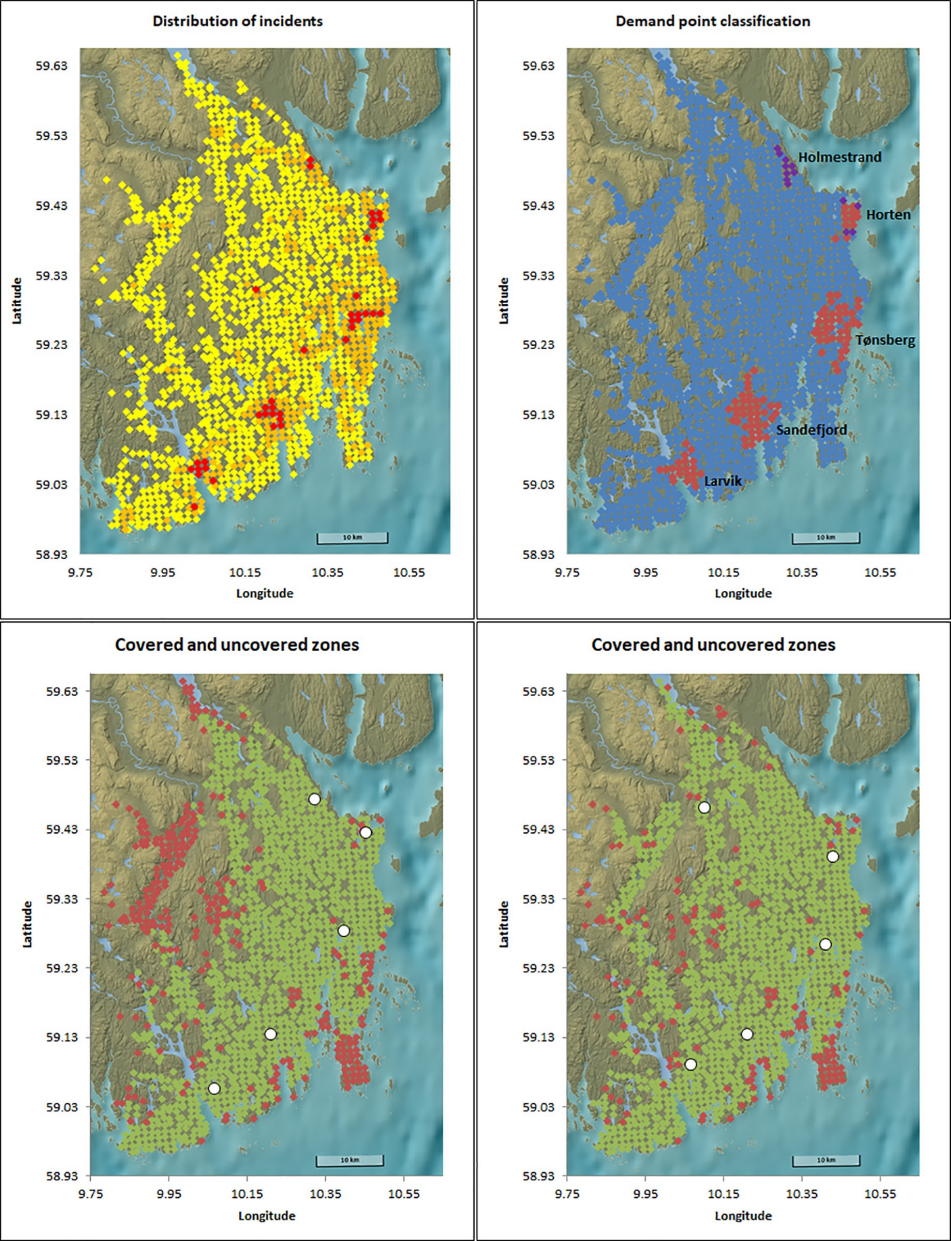
Upper left: Distribution of incidents (left), with demand points with more than 0.5% of all incidents in red. In orange are all demand points with number of incidents between 0.05% and 0.5% of all incidents. In yellow are demand points with less than 0.05% of all incidents. Upper right: Classification of demand points as urban (red) and rural (blue). In purple are the demand points that become urban when the cut-off for urban classification is decreased to 7000. Lower: Location of the five current bases (left) and the optimal five greenfield bases (right) using busy fraction 0. In green are covered areas, in red are uncovered areas. Background image reprinted from www.kartverket.no under a CC BY 4.0 license, with permission from Kartverket.

Population density and number of incidents are not necessarily highly correlated and using population data as a proxy for the expected number of incidents is not recommended [7]. In the analysis of optimal location of EMS bases, we use the number of ambulance assignments with the highest urgency level in the years from 2013 to 2016 as recorded by the Vestfold Hospital Trust as the expected incident intensity for each demand point. A total of 39 436 assignments were performed by the operating ambulances in the region, of which 37 754 have coordinates within the area under study. A further 32 of these were excluded because they arose from an unrouteable area. The geographical distribution of the ambulance assignments is shown in Fig 1.

For rigorous mathematical modeling, we divide the region into a set of demand points, using a grid of 1 km x 1 km squares throughout the region as provided by Statistics Norway [16]. County and municipality borders obtained from the Norwegian Mapping Authority [17] are used to extract the squares that are part of the service region. The region consists of 2261 squares of 1 km x 1 km. Excluding squares without any roads results in a total of 1972 squares. For each square, we then obtained the population from Statistics Norway [15] and the estimated number of incidents from the number of ambulance assignments between 2013 and 2016. In the calculations, only squares with either non-zero population or non-zero incidents were included as potential demand points from which requests can arise. This reduced the number of demand points to 1472 to be included in the analyses.

In order to optimize for the locations of the bases, the potential locations of these bases has to be specified. As even an uninhabited square can be a good location for a base, we include all 1972 routable 1 km x 1 km squares as potential base locations. Including also the exact locations of the current five bases and two standby points gives a total of 1979 potential base locations.

### Demand point classification

Each of the 1472 included demand points was classified as either urban or rural. This distinction is essential, as the official response time target is 12 minutes for urban areas and 25 minutes for rural areas [3]. Government recommendations state that a population center with more than 10 000 inhabitants is classified as urban [18]. A total of 116 demand points was classified as urban, distributed over four clusters surrounding the main population centers in the area (Fig 1). Notably, this governmental recommendation classifies the town of Holmestrand as a rural area. This is in contrast to how the inhabitants in the Vestfold region consider the area, and a response time of 25 minutes for Holmestrand is by health personnel considered unacceptable, and the region is currently classified as urban by the hospital trust. Based on this, we also explored the effect of decreasing the minimum number of inhabitants for a population area to be considered urban. Decreasing the cutoff from the official number of 10 000 to 7000 increased the number of urban demand points from 116 to 129, with 9 of the 13 additional urban demand points located in the Holmestrand area (Fig 1).

### Travel time data

To determine whether a potential base location can cover a certain demand point within the response time target, the response time from the base location to the demand point must be calculated. This response time consists of two factors: the actual travel time and the pre-trip delay. The pre-trip delay is the time between call initiation and the time the ambulance starts driving. From hospital log data, the median pre-trip delay for 2017 was 2:55 minutes, and we use 3 minutes as pre-trip delay in the calculations. With the response time targets of 12 and 25 minutes, this gives a maximum driving time of 9 minutes for urban areas and 22 minutes for rural areas.

For the travel time between a potential base location and a demand point, we use road data from the Norwegian Public Roads Administration [19]. The data contains pre-calculated driving times, based on the speed limits for each road. Based on the actual average travel speeds, calculated from data recorded in the central hospital registry for individual ambulance trips, we altered the speeds on certain roads to better reflect actual ambulance driving speeds. For roads with a speed limit of less than 40 km/h, we take the speed limit as actual speed. For roads with a speed limit of 40 km/h, we multiply the driving speed by 1.15. For speeds limits over 40 km/h, we multiply the driving speed by 1.20. For three specific main roads, we used different correction factors. We used 1.30 for Kirkeveien on Nøtterøy and 1.40 for both Lågendalsveien in Larvik and Riksvei 19 in Horten. For each square, we compute the nearest road to the center of the square. The pgr_dijkstraCost function of pgRouting [20] is used to compute the shortest distance and time between each potential base location and each demand point. This function is an implementation of Dijkstra’s shortest path algorithm [21].

### Mathematical modeling

A frequently used model for exploring base locations for EMS vehicles is the Maximum Covering Location Problem (MCLP) [4]. The MCLP model maximizes the number of inhabitants or incidents covered by at least one ambulance within a desired time by optimal allocation of a pre-defined number of facilities. The MCLP model does, however, only optimize the location of the base stations, lacking the necessary detail to establish the distribution of available ambulances among the bases. As such, the model represents a best case scenario, with an ambulance is always available at a base whenever needed. In order to incorporate the potential temporary unavailability of ambulances due to concurrent tasks a more advanced mathematical model is needed.

The busy fraction is the average fraction of time that an ambulance is unavailable. A generalization of the MCLP model that incorporates this busy fraction is the Maximum Expected Covering Location Problem (MEXCLP) [11]. The MEXCLP model simultaneously optimizes the location of bases *and* the distribution of ambulances so as to maximize the expected coverage, that is, the fraction of requests that can be reached within the given target time threshold. For a busy fraction of 0 the MEXCLP model is equivalent to the MCLP model. By adding the busy fraction to the model, backup coverage, that is, multiple ambulances covering the same area, becomes important. In general, the expected coverage of a demand area covered by *k* ambulances with a busy fraction of *q* is 1−*q^k^*. In the MEXCLP model, this fractional coverage is than weighted with the relative importance of a demand area. The number of inhabitants, or the number of incidents, in a certain demand area can be used as a measure for its relative importance. Note that, the incorporation of the busy fraction might lead to a different set of optimal base locations than using the simpler MCLP model.

In the original version of the MEXCLP model, the number of ambulances is fixed as part of the input to the model, but there is no restriction on the number of bases. While such an open-ended approach might be valuable for exploratory purposes, the model tends to lead to an overly high number of bases[14], often only marginally geographically separated. From a practical viewpoint this is often undesirable, and we have in the current work adapted the MEXCLP model slightly to account for this, adding a restriction on the number of bases so as to make results more practically feasible. As the original model considers a single response time target and the Vestfold region uses different targets for urban and rural areas, we further adapt the model to incorporate this distinction. S1 Appendix gives a precise mathematical description of the model that we used.

### Solution technique

The general MEXCLP model, as well as our slightly modified model, is formulated as an Integer Linear Programming problem [22]. This means that the objective value and the constraints are linear combinations of a set of integer variables. These models can be solved for reasonably sized instances in a short amount of time by solvers such as CPLEX [23] and Gurobi [24]. We implement the model in Java and call CPLEX providing us with an exact, mathematically optimal solution for each instance. The average computation time for each run was 43 seconds.

### Model variables

We compute the optimal configuration of bases and ambulances for multiple scenarios that differ with respect to three parameters: the number of ambulances, the number of bases, and the busy fraction.

For the number of ambulances, we consider all values from 1 through 15. Currently 13 ambulances are used during daytime and eight ambulances during nighttime in the region.

For the number and location of bases, we consider three cases of decreasing level of restrictions. First, we fix the bases to the current five. This gives us the optimal distribution of different numbers of ambulances over the existing bases. In order to explore the quality of the existing bases, in the second case, we allow a maximum of five bases, but remove the restriction of their location, considering all 1979 potential base locations. Finally, to show the potential gain of having an unlimited amount of bases, no restriction is put on the number of bases nor on their location in the third case. In all three cases, there is a restriction on the number of ambulances.

**Fig 2:**
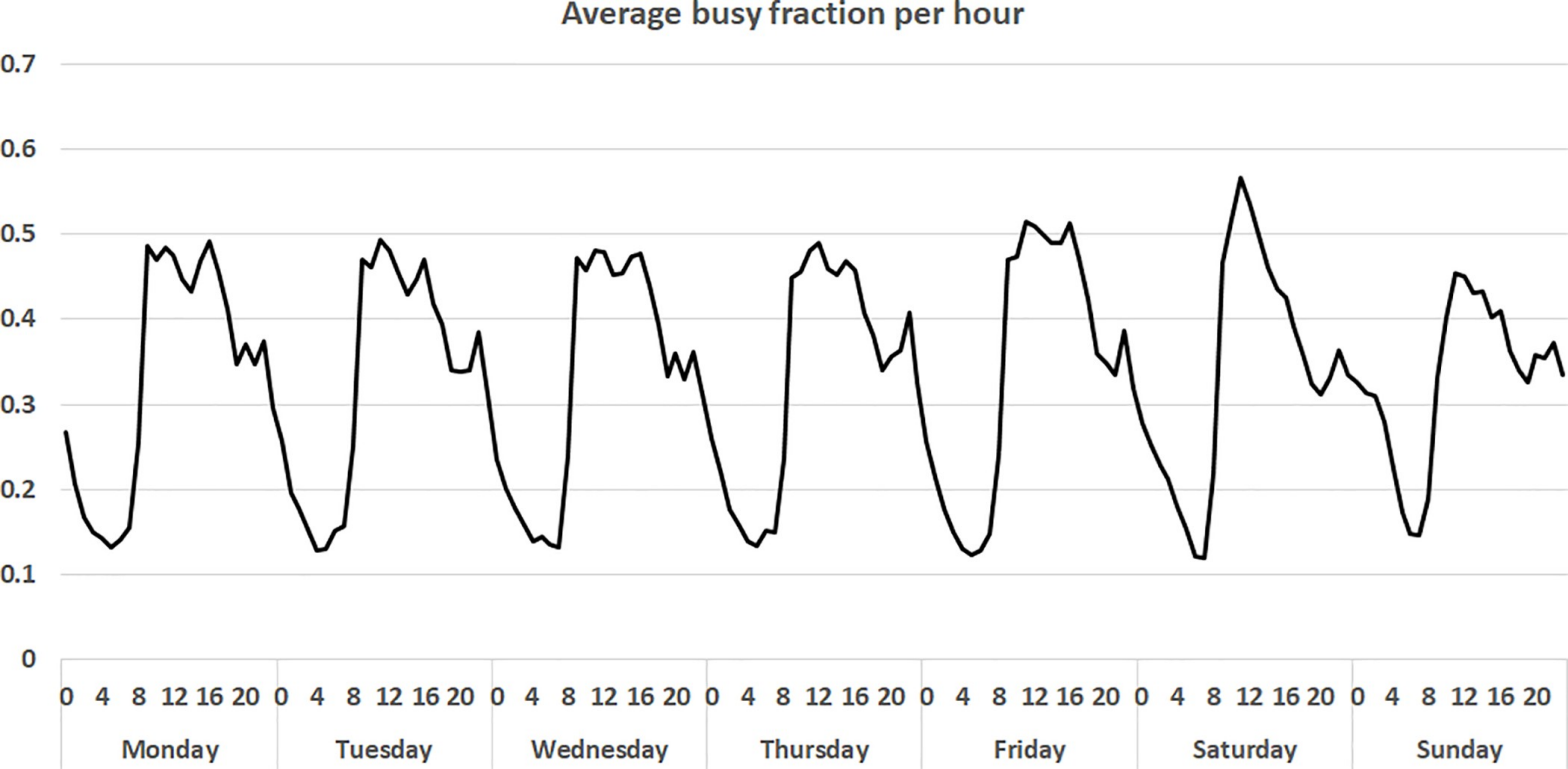
Average busy fraction per hour for different days, based on data from the years 2013–2016.

The busy fraction is the amount of time an ambulance is busy with a concurrent task whenever needed. The average busy fraction for the ambulances in the region changes throughout the day (Fig 2) and taking this fluctuation into accounting is important [14,25]. In the analyses, we consider the following four values for the busy fraction: 0, 0.15, 0.35, 0.50. The first case is equivalent to the MCLP model where only single coverage is important, while the last three values correspond with the average busy fraction during night, evening and day in the region (Fig 2). Note that varying the number of ambulances would also change the busy fraction. In this paper, we change the number of ambulances in order to show the relative importance of each ambulance. For day, evening and night, the number of ambulances corresponding with the busy fraction is 13, 10 and 8, respectively.

All possible combinations of the three parameters results in the fitting of a total of 180 scenarios. Finding the solution for all these scenarios allows us to explore the potential effect of changes in the included parameters.

## Results

### Single coverage

The MCLP model gives insight into optimal locations of base stations, regardless of the number of ambulances at each base. The five current bases have a total coverage of 93.46% of incidents when disregarding concurrent events (Table 1). The Tønsberg base alone provides a single coverage of 45.14%, with the Sandefjord, Larvik, Horten and Holmestrand bases successively adding a further 23.85%, 13.15%, 9.75% and 1.57% coverage, respectively. Comparably, the mathematical optimum using five bases in a greenfield scenario for single coverage increases coverage when using a maximum of five bases to 97.51% (Table 1). Notably, four of the current bases are geographically located close to the mathematical optimum (Fig 1). The optimal location of the fifth base, however, is significantly further west than the location of the current base.

**Table 1:** Single coverage given a fixed number of bases in the current situation and the greenfield situation.

*Number of bases*	*Coverage current bases*	*Coverage greenfield bases*
*1*	45.14%	46.96%
*2*	68.99%	78.24%
*3*	82.14%	90.26%
*4*	91.89%	95.84%
*5*	93.46%	97.51%
*6*		98.36%
*7*		98.55%
*15*		99.40%

### Multiple ambulances and bases

Using a nonzero busy fraction in the MEXCLP model, we adjust for concurrent events. For increasing values of the busy fraction, the focus of the model shifts from single coverage for low-demand areas to backup coverage and more on-call ambulances for high-demand areas. Table 2 gives the expected coverage when applying a busy fraction of 0.15, 0.35 and 0.5 for a fixed set of bases and for the optimal set of five bases, as well as the distribution of the additional ambulance in the current base structure.

**Table 2:** Expected coverage for different numbers of ambulances with a busy fraction of 0.15, 0.35 and 0.5 given the current bases and the restricted greenfield setting. Whenever an ambulance is added to the northernmost base, it is highlighted in bold.

	*Busy fraction = 0.15*			*Busy fraction = 0.35*			*Busy fraction = 0.5*		
Number of ambulances	Coverage current bases	Selected base location	Coverage restricted greenfield	Coverage current bases	Selected base location	Coverage restricted greenfield	Coverage current bases	Selected base location	Coverage restricted greenfield
*1*	38.37%	Tønsberg	39.92%	29.34%	Tønsberg	30.53%	22.57%	Tønsberg	23.48%
*2*	60.53%	Sandefjord	68.26%	48.22%	Sandefjord	54.21%	38.20%	Sandefjord	42.86%
*3*	72.56%	Larvik	80.04%	58.61%	Larvik	65.24%	47.64%	Tønsberg	53.48%
*4*	82.04%	Horten	86.04%	67.38%	Horten	73.20%	56.05%	Larvik	62.49%
*5*	85.05%	Tønsberg	89.22%	73.49%	Tønsberg	79.08%	62.40%	Horten	69.12%
*6*	87.51%	Sandefjord	92.13%	78.27%	Sandefjord	83.31%	68.17%	Sandefjord	73.72%
*7*	89.20%	Larvik	93.69%	81.44%	Larvik	86.36%	71.90%	Larvik	77.53%
*8*	**90.72%**	**Holmestrand**	94.71%	83.91%	Horten	88.49%	75.35%	Tønsberg	80.50%
*9*	91.84%	Horten	**95.83%**	85.76%	Tønsberg	90.30%	78.10%	Horten	83.50%
*10*	92.25%	Tønsberg	96.25%	87.29%	Sandefjord	91.66%	80.64%	Sandefjord	85.37%
*11*	92.60%	Sandefjord	96.55%	**88.45%**	**Holmestrand**	92.66%	82.38%	Larvik	87.04%
*12*	92.85%	Larvik	**96.84%**	89.52%	Larvik	93.36%	83.91%	Tønsberg	88.49%
*13*	**93.06%**	**Holmestrand**	97.09%	90.24%	Horten	**94.12%**	85.20%	Horten	89.92%
*14*	93.22%	Horten	97.26%	90.84%	Tønsberg	94.74%	86.40%	Sandefjord	90.77%
*15*	93.28%	Tønsberg	97.32%	91.36%	Sandefjord	95.27%	**87.31%**	**Holmestrand**	91.56%

Using the five current bases, we observe how a fifth available ambulance is not placed at the northernmost Holmestrand base: adding a second ambulance at the Tønsberg base, which covers a high-demand area, adds more coverage than the first ambulance in Holmestrand. Indeed, the higher the busy fraction, the later it becomes beneficial to locate an ambulance at the northernmost base, but rather focus on the urban areas in the southern part of the region. For busy fractions 0.15, 0.35 and 0.5 only the eighth, eleventh, or fifteenth available ambulance, respectively, is located in Holmestrand.

When allowing the MEXCLP model to freely select the optimal location of five bases, we see similar behavior. In case of a busy fraction of 0.15, only with nine available ambulances, a base will be opened in the North. With fewer ambulances, an additional base in the Tønsberg area is preferred. Note that the five selected bases are equivalent to the MCLP bases. For a busy fraction of 0.35 or 0.5, the northern base is only used when 13 or 19 ambulances are available.

### Greenfield analyses

**Fig 3:**
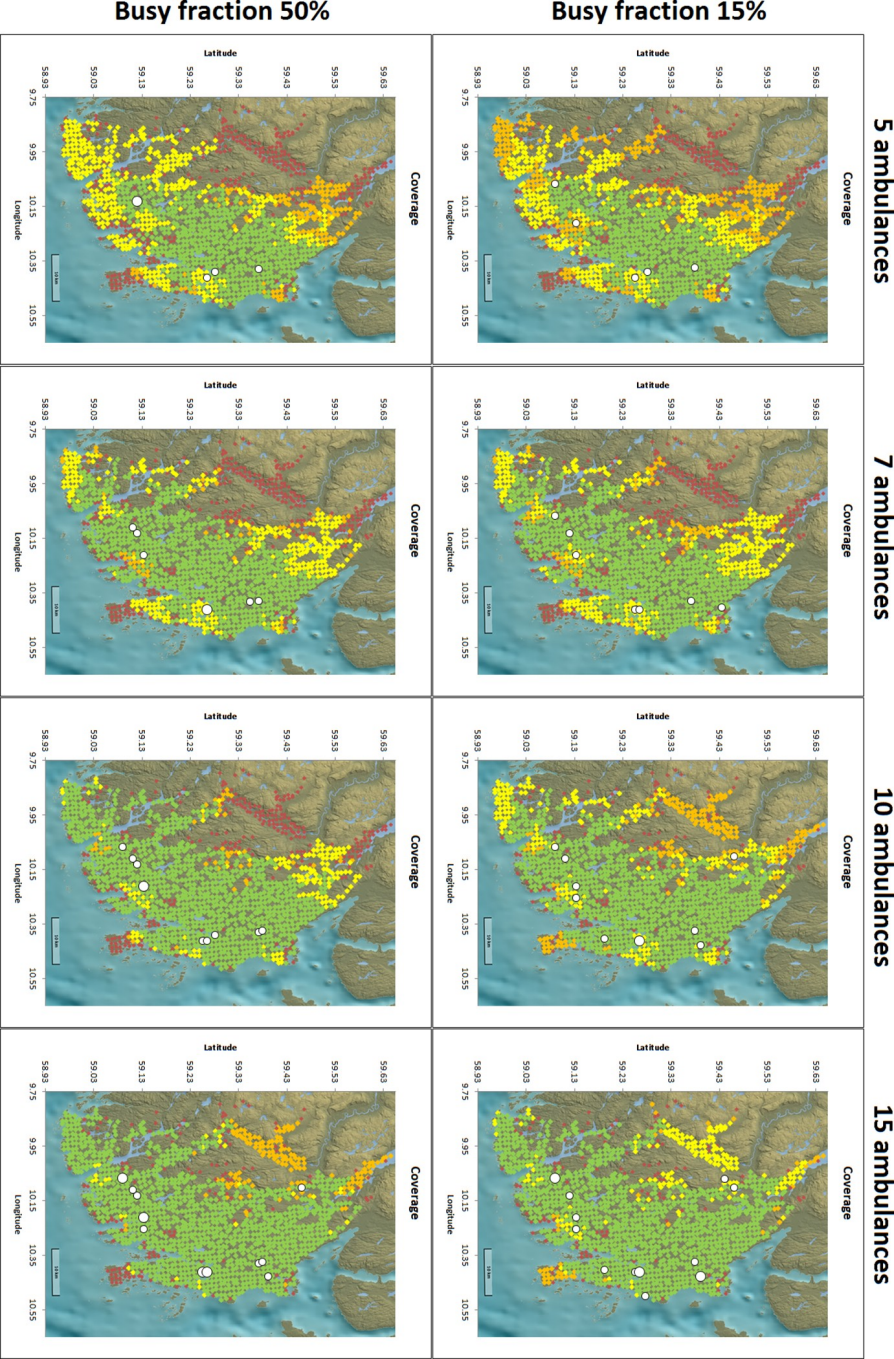
Locations for busy fractions and number of ambulances in greenfield analyses. Green represents triple coverage, yellow double coverage, orange single coverage, and red uncovered. A larger white circle indicates two ambulances at the same base location. Background image reprinted from www.kartverket.no under a CC BY 4.0 license, with permission from Kartverket.

Placing no restriction on neither the number of bases, their location, nor the number of ambulances per base in a complete greenfield scenario, the structure of the optimal solution is generally to have one ambulance per base, and spread the bases throughout the region (Fig 3). Interestingly, the bases from such a greenfield scenario typically replace the bases from the scenario with a maximum of five bases by small clusters of bases around the originally selected bases. The overall coverage in the greenfield scenario is slightly higher compared to fewer bases with multiple ambulances (Table 3). In this scenario, with busy fractions 0.15, 0.35 and 0.5, the northernmost base is used by the eighth, eleventh and fourteenth ambulance, respectively.

**Table 3:** Expected coverage for different numbers of ambulances with a busy fraction of 0.15, 0.35 and 0.5 given the unrestricted and restricted greenfield setting. Whenever an ambulance is added to the northernmost base, it is highlighted in bold.

	*Busy fraction = 0.15*		*Busy fraction = 0.35*		*Busy fraction = 0.5*	
Number of ambulances	Coverage restricted greenfield	Coverage greenfield	Coverage restricted greenfield	Coverage greenfield	Coverage restricted greenfield	Coverage greenfield
*1*	39.92%	39.92%	30.53%	30.53%	23.48%	23.48%
*2*	68.26%	68.26%	54.21%	54.21%	42.86%	42.86%
*3*	80.04%	80.04%	65.24%	65.24%	53.48%	53.48%
*4*	86.04%	86.04%	73.20%	73.20%	62.49%	62.49%
*5*	89.22%	89.22%	79.08%	79.08%	69.12%	69.12%
*6*	92.13%	92.33%	83.31%	83.34%	73.72%	73.77%
*7*	93.69%	94.20%	86.36%	86.47%	77.53%	77.69%
*8*	94.71%	**95.44%**	88.49%	88.65%	80.50%	80.74%
*9*	**95.83%**	96.25%	90.30%	90.65%	83.50%	83.60%
*10*	96.25%	96.86%	91.66%	91.97%	85.37%	85.53%
*11*	96.55%	97.31%	92.66%	**93.19%**	87.04%	87.42%
*12*	**96.84%**	97.67%	93.36%	94.08%	88.49%	88.84%
*13*	97.09%	97.90%	**94.12%**	94.80%	89.92%	90.19%
*14*	97.26%	**98.08%**	94.74%	95.52%	90.77%	**91.12%**
*15*	97.32%	98.24%	95.27%	96.00%	91.56%	92.04%

### Urban/Rural alternative

**Fig 4:**
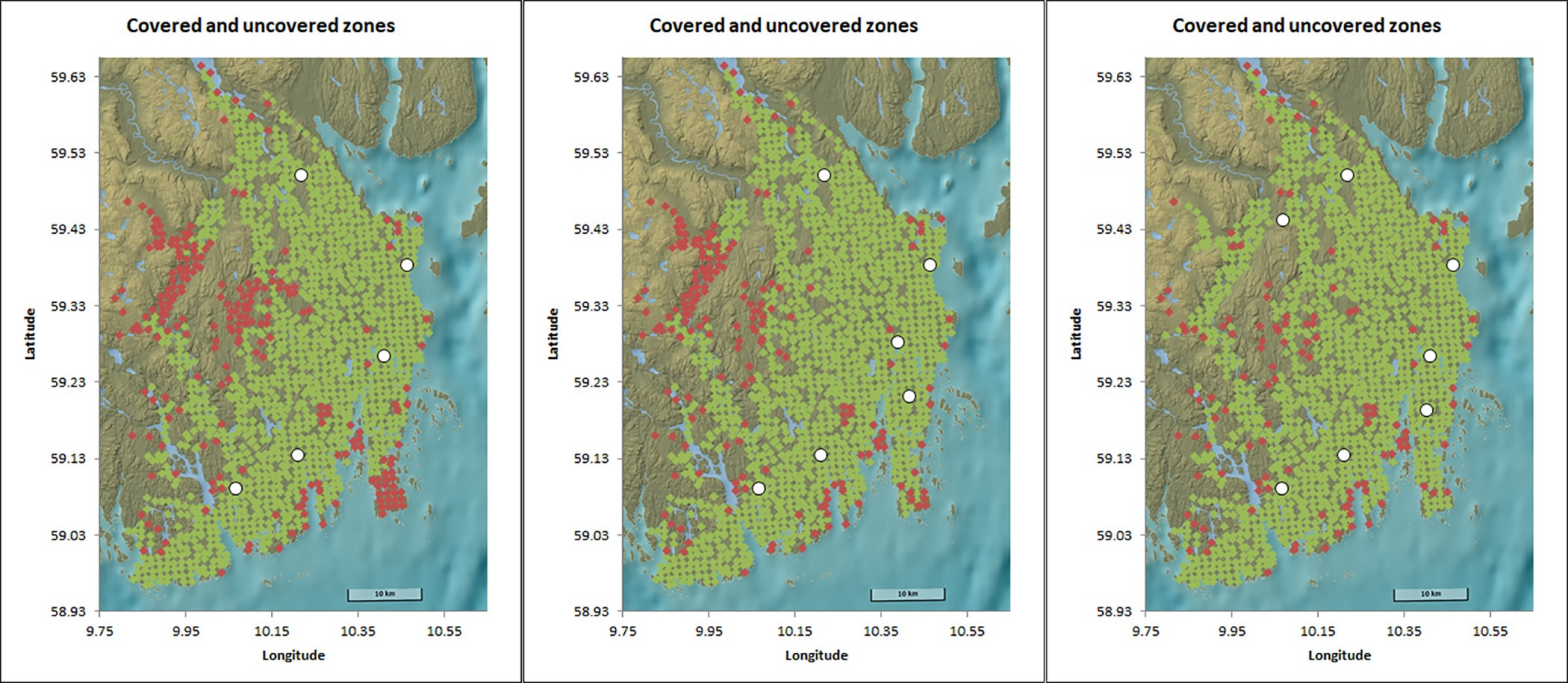
Locations of the bases in the greenfield analyses with Holmestrand as an urban area and 5, 6 and 7 bases. Green represents a covered and red an uncovered area. Background image reprinted from www.kartverket.no under a CC BY 4.0 license, with permission from Kartverket.

Decreasing the cut-off for classification of a demand area from rural to urban from 10 000 to 7000 changes the results markedly. Using the MCLP the optimal location of the fifth base is now significantly closer to Holmestrand, though yet still somewhat further west than the current location of the base (Fig 4). Increasing the number of bases, the sixth base would be used to improve coverage in the surroundings of the main city Tønsberg, and the seventh base to cover the large rural areas in the northwest.

Using the current base structure and a busy fraction of 0.15 the fifth ambulance is now located at the Holmestrand base to ensure coverage within 9 minutes, in contrast to when using the official guidelines and a cutoff of 10 000 inhabitants. Increasing the busy fraction, the second ambulance in Tønsberg does still add more coverage than the first in Holmestrand and only the seventh or eleventh ambulance is located in Holmestrand. In the restricted greenfield setting, only the sixth, tenth, or fourteenth ambulance is located near Holmestrand for a busy fraction of 0.15, 0.35, and 0.5. Only in a complete greenfield scenario an additional base is opened to cover the large rural area in the northwest, provided that enough ambulances are available.

## Discussion

The five existing EMS bases in the Vestfold region match the five geographical clusters with a high number of incidents. Disregarding concurrent events, mathematical modeling demonstrates that four of the five current bases are geographically well located to provide adequate service. However, as most of the Holmestrand area, a town in the Northern part of the region, can be covered by a nearby base, moving this northernmost base further west, in order to cover a large, currently uncovered, rural area, would increase coverage. In combination with a slight adjustment of the other four bases, overall coverage could be increased by up to 4 percentage points without increasing neither the number of bases nor the number of ambulances.

Including concurrent events in the model, and increasing the corresponding busy fraction, results remain consistent. In all mathematical models the five optimal base locations are similar to the greenfield scenario of the best case scenario MCLP model. Even when allowing for more than five bases in the models, the additional bases tend to cluster around the original five base locations (Fig 3). The main difference when changing the busy fraction in the model is the total number of ambulances required to allocate one to the fifth base. In all cases with a non-zero busy fraction, the fifth available ambulance is not placed at this fifth base location, but rather used as a second ambulance in the largest city in the region, Tønsberg. For increasing values of the busy fraction, the number of required ambulances increases. Only when sufficiently many ambulances are available, it is beneficial to move one ambulance in order to cover the large rural area in the Northern part of the region. These results could be implemented in everyday practice by opening a temporary base in the North that only has an ambulance whenever enough ambulances are available in the region.

The mathematical model applied in this study optimizes the expected coverage regarding the two response time targets. It does however not take the average response time into account. Moving the northernmost base from the more urban Holmestrand region towards the less populated region to the West will increase the response time to incidents in the Holmestrand area. While this will cause the expected coverage within the target times to increase, it will also result in an increase in the average response time for all events. Focusing solely on one objective, such as the incident coverage within the response time target, like in this study, will generally lead to suboptimal solutions for other objectives, such as the average response time. In future studies it would be interesting to include the average response time as part of the objective to explore potential impact of the optimal location of bases.

While all mathematical models pointed to moving the Holmestrand base further west than the current location of the base, it was deemed unacceptable by Vestfold hospital trust that the Holmestrand area would experience increased response times. Using the classification from the Ministry of Social Affairs and Health, the region is viewed as rural. Rerunning the analysis with an alternative definition of urbanity resulted in the fifth base being located significantly closer to Holmestrand, and more in line with the perception of both inhabitants and health personnel in the region. This highlights the importance of critically reviewing data used in a mathematical model. Using governmental guidelines as input might not optimally reflect the situation under study. A close collaboration between mathematicians and practitioners is essential when analyzing potential improvements to the location of ambulance bases and the distribution of available ambulances.

## Conclusion

Emergency medical service providers are responsible for providing quick responses in emergency situations. In regions with both urban and rural areas, a trade-off must typically be made between single coverage for low-demand areas and back-up coverage for high-demand areas. This study shows how this trade-off depends on the load of the system and the number of available ambulances. If the busy fraction, and thus the load on the system, increases, focus should be shifted from low-demand to high-demand areas. Furthermore, this study shows that the right classification of demand points is important and that national guidelines might not be optimal. This demonstrates the importance for a close collaboration between mathematicians and practitioners.

## Supporting information

S1 Appendix Model formulation. This file contains a precise mathematical formulation of the model.
(DOCX)

S1 File List of demand points. Data includes the id, longitude, latitude, population and number of incidents for each demand point.
(CSV)

S2 File List of current base stations. The file contains the id, name, type, longitude, latitude for each current demand location. The type can be current station or standby point.
(CSV)

S3 File Busy fraction data. The file contains the number of ambulances on duty and the number of busy ambulances for each hour over the years 2013–2016.
(CSV)
