# Supplementary material for: Improving ambulance coverage in a mixed urban-rural region in Norway using mathematical modeling
Source: PLoS One. 2019 Apr 12;14(4):e0215385. doi: 10.1371/journal.pone.0215385 (PMC6461285; doi:10.1371/journal.pone.0215385)
Supplement: S1 Appendix — This file contains a precise mathematical formulation of the model. (DOCX) [file pone.0215385.s001.docx]

**Appendix 1**

This appendix gives a complete overview of the mathematical model that has been used in this paper by introducing the used sets, parameters, decision variables, objective and constraints. The model is a slightly modified version of the Maximum Expected Covering Location Problem [1].

**Sets**

$I$ = set of potential base locations

$J$ = set of demand areas

**Parameters**

$d_{j}$ = relative weight of demand area $j$. In our case, this is based on the number of incidents between 2013 and 2016.

$q$ = average busy fraction

$p$ = maximum number of ambulances that can be located

$b$ = maximum number of bases that can be opened

$\tau_{j}$ = response time target for demand area $j$. In our case, $\tau_{j}$ = 12 for urban areas and $\tau_{j}$ = 22 for rural areas.

$t_{ij}$ = travel time between potential base location $i$ and demand area $j$, excluding the pre-trip delay.

$M$ = sufficiently large constant. In our case, $M=p$ is sufficient.

**Decision variables**

$x_{i}= the number of ambulances located at potential base location i$

$$y_{jk}= \left\{ \begin{aligned} 1 if demand area j is covered by at least k ambulances within its response time \\ \mathrm{target}\tau_{j} \\ 0 otherwise \end{aligned} \right.$$

$$z_{i}= \left\{ \begin{aligned} 1 if at least one ambulances is located at potential base location i \\ 0 \mathrm{otherwise} \end{aligned} \right.$$

**Objective**

The objective is to maximize the expected coverage.

$$\max\sum_{j\in J} \sum_{k=1}^{p} d_{j}\left( 1-q \right)q^{k}y_{jk}$$

**Constraints**

The following constraint ensures that the $y$-variables have the correct value, given the values for the $x$-variables.

$$\sum_{\{i\in I: t_{ij} \leq\tau_{j}\}} x_{i}\geq\sum_{k=1}^{p} y_{jk} \forall j\in J$$

Next, we restrict the total number of ambulances by $p.$

$$\sum_{i\in I} x_{i}\leq p$$

Similarly, we restrict the total number of opened bases by $b.$

$$\sum_{i\in I} z_{i}\leq b$$

To ensure that the $z$-variables have the correct value, based on the value of the $x$-variables, we add the following restrictions.

$x_{i}\leq M\times z_{i} \forall i\in I$

Finally, we have the domain restrictions for the variables.

$x_{i}\in\left\{ 0, 1, 2, \ldots, p \right\}$

$y_{jk}\in\left\{ 0,1 \right\}$

$z_{i}\in\{0,1\}$

**References**

1. Daskin MS. A Maximum Expected Covering Location Model: Formulation, Properties and Heuristic Solution. Transportation Science. 1983;17(1): 48–70.
